# Clinical motion analyses over eight consecutive years in a child with crouch gait: a case report

**DOI:** 10.1186/s13256-016-0920-9

**Published:** 2016-06-15

**Authors:** Erin E. Butler, Katherine M. Steele, Leslie Torburn, James G. Gamble, Jessica Rose

**Affiliations:** 1 The William H. Neukom Institute for Computational Science, Dartmouth College, Sudikoff Hall, Hanover, NH 03755 USA; 2 Department of Mechanical Engineering, University of Washington, 3600 E Stevens Way NE, Box 352600, Seattle, WA 98195 USA; 3 Motion & Gait Analysis Laboratory, Lucile Packard Children’s Hospital at Stanford, 321 Middlefield Road, Menlo Park, CA 94025 USA; 4 Department of Orthopaedic Surgery, Stanford University School of Medicine, 770 Welch Road, Suite 400, Palo Alto, CA 94304 USA

**Keywords:** Cerebral palsy, Crouch gait, Clinical motion analysis, Outcomes, Botulinum toxin-A

## Abstract

**Background:**

This case report provides a unique look at the progression of crouch gait in a child with cerebral palsy over an 8-year time period, through annual physical examinations, three-dimensional gait analyses, and evaluation of postural balance. Our patient received regular botulinum toxin-A injections, casting, and physical therapy but no surgical interventions.

**Case presentation:**

A white American boy with spastic diplegic cerebral palsy was evaluated annually by clinical motion analyses, including physical examination, joint kinematics, electromyography, energy expenditure, and standing postural balance tests, from 6 to 13 years of age. These analyses revealed that the biomechanical factors contributing to our patient’s crouch gait were weak plantar flexors, short and spastic hamstrings, moderately short hip flexors, and external rotation of the tibiae. Despite annual recommendations for surgical lengthening of the hamstrings, the family opted for non-surgical treatment through botulinum toxin-A injections, casting, and exercise. Our patient’s crouch gait improved between ages 6 and 9, then worsened at age 10, concurrent with his greatest body mass index, increased plantar flexor weakness, increased standing postural sway, slowest normalized walking speed, and greatest walking energy expenditure. Although our patient’s maximum knee extension in stance improved by 14 degrees at 13 years of age compared to 6 years of age, peak knee flexion in swing declined, his ankles became more dorsiflexed, his hips became more internally rotated, and his tibiae became more externally rotated. From 6 to 9 years of age, our patient’s minimum stance-phase knee flexion varied in an inverse relationship with his body mass index; from 10 to 13 years of age, changes in his minimum stance-phase knee flexion paralleled changes in his body mass index.

**Conclusions:**

The motor deficits of weakness, spasticity, shortened muscle-tendon lengths, and impaired selective motor control were highlighted by our patient’s clinical motion analyses. Overall, our patient’s crouch gait improved mildly with aggressive non-operative management and a supportive family dedicated to regular home exercise. The annual clinical motion analyses identified changes in motor deficits that were associated with changes in the child’s walking pattern, suggesting that these analyses can serve to track the progression of children with spastic cerebral palsy.

## Background

Crouch gait is one of the most common gait disorders among children with spastic cerebral palsy (CP) [1]. It is characterized by excessive flexion of the hip, knee, and ankle during stance [2]. The specific causes of crouch gait vary among individuals, but the primary biomechanical contributors include short and/or spastic hamstrings [3], short hip flexors [4, 5], weak hip and knee extensors [6], weak ankle plantar flexors [7], and/or malrotation of the femur, tibia, and foot [8, 9]. Poor selective motor control and poor balance also contribute to gait deficits [10, 11]. With each progressive degree of knee flexion during stance there is a proportional increase in the demand on the knee extensors [12]. Thus, individuals with CP and crouch gait commonly exert more energy while walking than their peers [13, 14] and are at an increased risk for joint pain and degeneration, formation of bony deformities, and loss of independent gait [15–18].

It has been posited that crouch gait worsens with age due to increased body size and weight [14, 19, 20]. However, few studies have documented the progression of gait with age among children with CP treated non-surgically [20–23], and even these studies did not focus specifically on crouch gait and were limited to two time points. The purpose of this paper was to report the progression of crouch gait with non-surgical treatment through clinical motion analysis, which included joint kinematics, surface electromyography (EMG), and energy efficiency during gait, physical examination, and postural balance, in a single individual over 8 years. We further aimed to highlight the contributions of the motor deficits associated with spastic CP (that is, muscle weakness, shortened muscle-tendon lengths, spasticity, and impaired selective motor control) to crouch gait.

## Case presentation

A 6-year-old white American boy with a diagnosis of spastic bilateral CP, Gross Motor Function Classification System (GMFCS) [24] level I was referred to our clinical motion analysis laboratory. Our patient was born full term, with no history of epilepsy and no noted disturbances of sensation, perception, cognition, communication, or behavior. He received annual evaluations in the same laboratory for 8 years from ages 6 to 13 years. At each visit a physical examination and video analysis were performed, and our patient underwent instrumented three-dimensional gait analysis, including kinematics and dynamic surface EMG, as well as postural standing balance tests and analysis of his energy efficiency during gait. His treatment schedule of botulinum toxin-A injections (BoNT-A) and casting are displayed in Table 1. Our patient was referred and treated by a physician at an outside hospital.

**Table 1:** Treatments received by our patient and the age at which treatments were administered

Treatment		Age (years)													
		6	6.5	7	7.5	8	8.5	9	9.5	10	10.5	11	11.5	12	12.5
BoNT-A	Hamstrings (bilateral)	*		*		*		*		*	*	*	*	*	*
	Gastrocnemius (right)	*		*		*		*		*	*	*	*	*	*
	Gastrocnemius (left)	*		*									*		*
	Psoas (bilateral)										*		*		
	Rectus femoris (right)											*			
Casting	Long leg (bilateral)			*		*		*			*	*	*	*	*
	Serial (right ankle)	*		*			*								

*BoNT-A* botulinum toxin-A injections

Treatments listed for the whole year were administered 2–10 weeks after the annual gait analysis was performed. Treatments listed for the half year occurred at least 3 months prior to the subsequent gait analysis. For example, a gait analysis was performed at age 8; less than 10 weeks after this gait analysis, our patient received BoNT-A injections to his bilateral hamstrings and right gastrocnemius with long leg casting; approximately 6 months later at age 8.5 years, our patient had serial casting at his right ankle. Our patient received 300 units of BoNT-A at each treatment age, with the exception of age 10.5 years when he received 200 units of BoNT-A. The BoNT-A injections and long leg casting were performed under general anesthesia.

During the study interval, our patient participated in a wide variety of sports, including tennis, swimming, skiing, and horseback riding. He occasionally wore ankle-foot orthoses. His family reported he had an intensive home exercise program of stretching and strengthening exercises for the trunk and legs, as instructed in physical therapy; his father helped him with the exercise program to facilitate adherence.

### Assessments

At each visit to the clinical motion analysis laboratory, our patient’s body mass index (BMI) was calculated. Because BMI is both age-specific and sex-specific for children, the BMI-for-age percentile for boys was used to interpret his BMI [25]. His leg length was measured supine from the anterior superior iliac spine to the medial malleolus. Hip, knee, and ankle passive range of motion (ROM) measures were recorded and muscle strength was measured using the six-point (0–5) Manual Muscle Test [26, 27]. At age 13 years, our patient’s selective motor control was tested using the recently introduced Selective Control Assessment of the Lower Extremity (SCALE) tool [28].

Three-dimensional kinematics were collected during barefoot walking with an eight-camera optoelectric system (Motion Analysis Corporation, Santa Rosa, CA, USA). The Gait Deviation Index (GDI) was calculated from lower extremity kinematics [29], and his medial hamstrings’ muscle-tendon length and lengthening velocity were calculated using SIMM (MusculoGraphics, Inc., Chicago, IL, USA) [30]. Surface EMG was collected for his bilateral rectus femoris, lateral quadriceps, medial hamstrings, medial gastrocnemius, and tibialis anterior using the MA-200 EMG system (Motion Lab Systems, Inc., Baton Rouge, LA, USA). EMG and foot switch data were processed using EMG Analyzer software (B&L Engineering, Santa Ana, CA, USA).

Standing postural balance measures of center of pressure path length and average radial displacement were collected, as previously reported [31]. The energy efficiency index (EEI) was recorded during a 2-minute walk at a patient-selected comfortable walking speed and measured in heartbeats per meter walked, as previously reported [32].

The same experienced physical therapist and bioengineer conducted the clinical motion analysis every year. After each session, the results were presented to a multidisciplinary clinical team and recommendations for treatment were made. Treatment recommendations were then sent to our patient’s referring physician at an outside hospital. Our patient’s parents provided informed consent for the presentation of data for scientific publication. The Institutional Review Board deemed the report exempt from review and approval, as the report did not meet the definition of “Human Subjects Research.”

### Outcomes

#### Physical examination

**Fig. 1:**
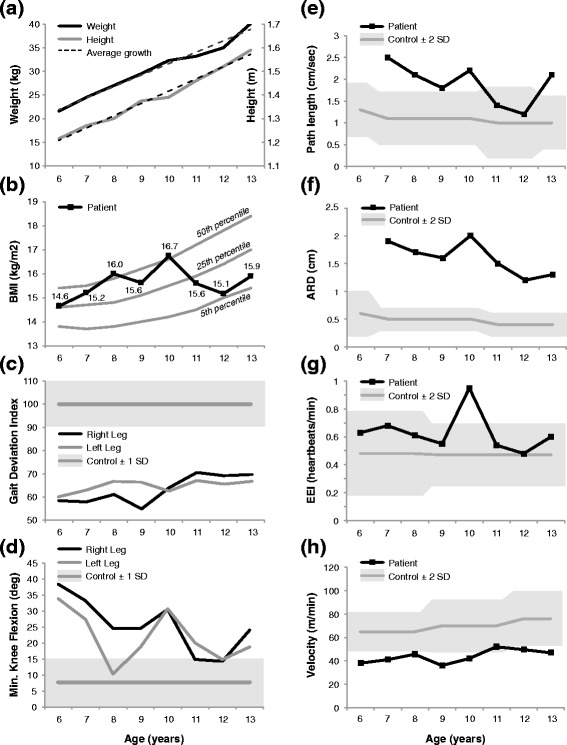
Key outcome measures. **a** Height and weight with the average growth rate indicated by *dashed lines*; **b** body mass index (*BMI*), with the 50th, 25th, and 5th BMI-for-age percentiles for boys; **c** the Gait Deviation Index; and **d** maximum stance-phase knee extension. Postural balance measures of **e** path length and **f** average radial displacement (*ARD*), during the eyes open testing condition. Path length represents the distance traveled by the center of pressure centroid per second, while ARD represents the radial deviation of the center of pressure centroid relative to the mean centroid location. **g** Energy expenditure as measured by the energy expenditure index (*EEI*) during a 2-minute walking test; and **h** self-selected walking speed. All values are presented for ages 6 to 13 years

Our patient’s height and weight increased linearly at an average rate of 5.2 cm/year and 2.4 kg/year, respectively, which is slightly below the average rate of height and weight growth for a typically developing male in the USA [33]. His BMI remained within the range of 14.6 to 16.7 kg/m^2^ (Fig. 1a, b), peaking at age 10.

Notable ROM measures, including any muscle contractures, are listed in Table 2. All muscles tested were strong (5/5), excepting the hip abductors (4/5) and ankle plantar flexors (3/5 for ages 6–8 years and 2/5 for ages 9–13 years).

**Table 2:** Notable passive range of motion measures for ages 6–13 years, with exceptions noted

Passive range of motion	Right	Left
Hip extension	10–20° flexion^a^	10–20° flexion^a^
Hip internal rotation/external rotation	70–75°/45–70°	65–80°/40–55°^b^
Hip abduction	30–45°	20–40°
Popliteal angle (full knee extension = 0°)	65–80°	60–75°
Knee extension	5–10° flexion	0–5° hyperextension^c^
Ankle dorsiflexion (knee extended)	0–10°	0–10°
Ankle dorsiflexion (knee flexed)	5–20°	5–20°

^a^There were 0° hip flexion contractures, bilaterally, at age 11 years

^b^External hip rotation on the left was limited to 25° at age 13 years

^c^There was a 10° knee flexion contracture on the left at ages 6 and 7 years

Based on results of the SCALE at age 13, selective motor control was moderately impaired on his right (5 out of 10 possible points) and mildly impaired on his left (8 out of 10 points). Selective motor control was normal for his right hip and left hip, knee, and ankle. His right knee, ankle, and subtalar joints and left subtalar and toe joints had impaired selective motor control, while his right toe joints had no selective motor control.

#### Gait analysis: temporal-spatial parameters, kinematics, and electromyography

His mean GDI improved from 59 to 68 between ages 6 and 13 (Fig. 1c) but remained well below the mean for unimpaired gait (mean 100, SD 10).

From 6 to 13 years of age, his stride length (normalized to leg length) decreased from 1.29 to 1.18, cadence decreased from 131 to 111 steps/min, and single-limb support on the right decreased from 42.4 % of the gait cycle at age 6 to 36.2 % at age 13. Single-limb support on his left remained relatively constant. His step width normalized to leg length was greatest at age 6 (0.25), while double-limb support was greatest at age 13 (24 % of the gait cycle).

**Fig. 2:**
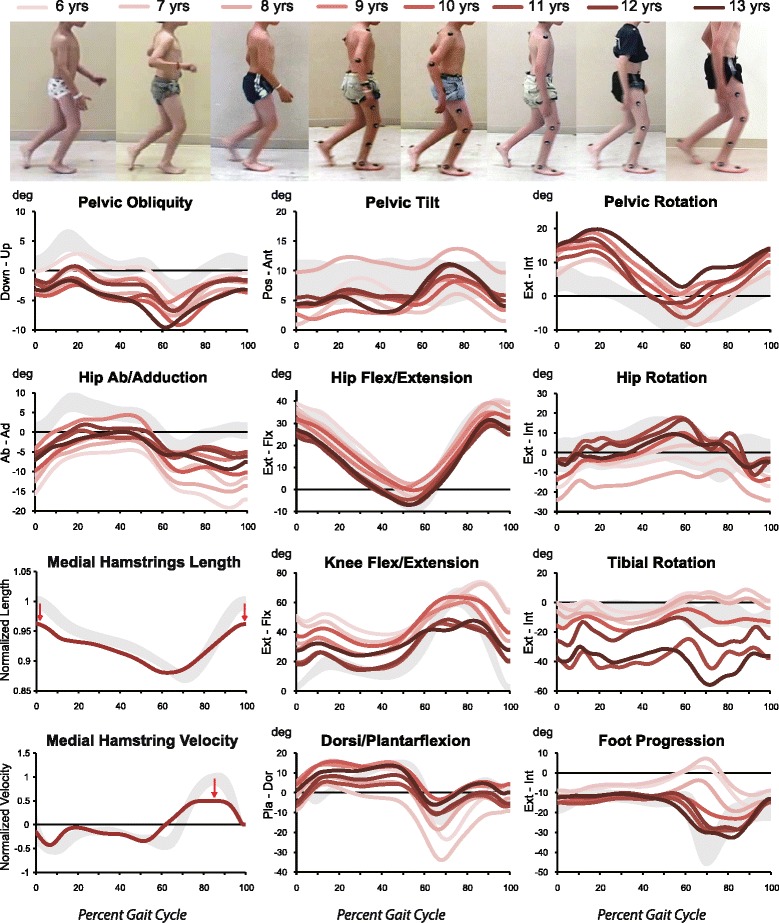
Right-side kinematics based on three-dimensional gait analysis. Joint kinematics are displayed for ages 6–13 years, including representative biomechanical analysis of medial hamstring length and lengthening velocity during gait

Our patient’s gait kinematics are displayed in Fig. 2 for his right side. Gait kinematics on his left were similar, though slightly less impaired than those on his right. Although our patient’s gait kinematics varied over the years, there were general kinematic patterns, as outlined below:

- Pelvis: His pelvis was held in a posture of rotation to the left with left upward pelvic obliquity throughout the gait cycle. There was “double-bump” pelvic anteversion during the gait cycle.
- Hip: Hip flexion and extension were nearly within normal limits (WNL) through the gait cycle. His right hip rotated internally during stance. His left hip was internally rotated through the gait cycle, secondary to the retracted pelvis on the ipsilateral side. His right hip was abducted and his left hip was adducted with respect to the oblique pelvis.
- Knee: At initial contact, his knees were flexed, right more so than left. There was reduced knee extension in midstance, right more so than left. Peak knee flexion during swing was reduced on his left, and reduced and delayed on his right. His tibiae were externally rotated.
- Ankle: His ankles were slightly plantar flexed at initial contact, with peak dorsiflexion generally occurring in loading response. His right foot progression angle was WNL, while his left foot progression angle was slightly internally rotated during stance.

His maximum stance-phase knee extension for ages 6–13 years is displayed in Fig. 1d. In general, his maximum knee extension in stance improved from 6 to 9 years, worsened at age 10, improved from 11 to 12 years, and worsened again at age 13.

Peak knee flexion in swing declined gradually from 6 to 13 years (Fig. 2): on the right from 70° to 45°, and on the left from 62° to 51°, with the most notable decline occurring after age 10. His hips became slightly more internally rotated, his tibiae became more externally rotated, and his ankles became more dorsiflexed in stance from 6 to 13 years (Fig. 2). His foot progression angle remained 10–15° externally rotated from 6 to 13 years of age, bilaterally.

Analysis of his muscle-tendon lengths and lengthening velocities of his medial hamstrings suggest his hamstrings were consistently short at initial contact and had a reduced lengthening velocity in swing (Fig. 2).

**Fig. 3:**
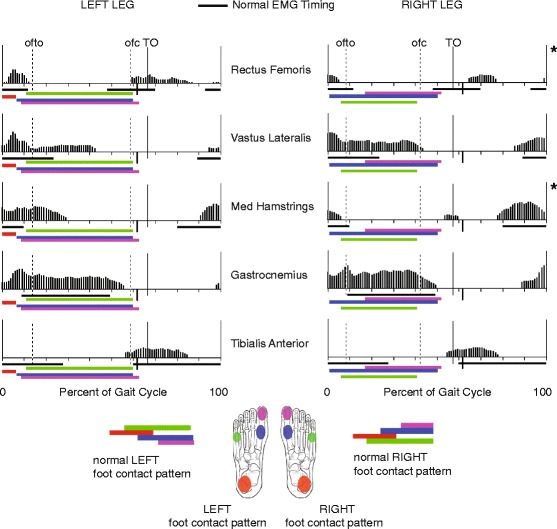
Representative electromyography (*EMG*) profiles and foot contact patterns. EMG profiles are normalized to the gait cycle. Exceptions to the typical patterns are noted with an asterisk: his right rectus femoris varied from continuously active, to active from initial swing through midswing and in loading response (as shown); his right medial hamstrings had continuous activity at ages 6 and 7 years, following which it was active from terminal swing through loading response with additional activity from preswing to initial swing (as shown). *ofto* opposite foot toe-off, *ofc* opposite foot contact, *TO* toe-off

His EMG profiles during gait varied little over time, with the exception of his right rectus femoris and right medial hamstrings (see Fig. 3 for details). His rectus femoris had prolonged activity in midswing, and his vastus lateralis and medial hamstrings had increased activity in stance. There was premature onset of his gastrocnemius in terminal swing and premature cessation of his tibialis anterior in midswing, bilaterally.

#### Postural balance

His center of pressure path length with the eyes open was greater than age-matched reference values [31] from 7 to 10 years of age, and again at age 13 (Fig. 1e). The average radial displacement remained consistently greater than two standard deviations above reference values, peaking at age 10 (Fig. 1f). Our patient was unable to comply with balance testing procedures at age 6. In general, his postural balance measures improved from 7 to 9 years, worsened at age 10, improved from 11 to 12 years, and worsened again at age 13.

#### Energy efficiency

His EEI was WNL every year with the exception of age 10, when his EEI was more than two standard deviations above the mean (Fig. 1g). There was no change in EEI at age 6 versus age 13. His comfortable walking speed was at least two standard deviations slower than age-matched reference values [32] each year, with the exception of age 11 when his walking speed was just WNL (Fig. 1h). When normalized to leg length, his comfortable walking speed decreased overall from 6 to 13 years of age. In general, the energy efficiency measures improved from 7 to 9 years, worsened at age 10, improved from 11 to 12 years, and worsened again at age 13.

#### Treatment recommendations

Based on the results of the gait evaluation, the multidisciplinary team recommended BoNT-A injections to his hamstrings and calf after the first motion analysis at age 6 years, and surgical hamstring lengthenings, as well as physical therapy for strengthening and balance training, annually thereafter. The reduced hamstring length and lengthening velocity, generated from the gait kinematic data, were most indicative of the need for hamstring lengthenings. However, his family opted against surgical intervention during the study period, and it was our patient’s family and his referring physician (at an unaffiliated hospital) who determined which treatments were performed. The schedule of BoNT-A and casting treatments are displayed in Table 1. Our patient’s parents reported that he tolerated the treatments well, was compliant in each 2-week session of post-intervention physical therapy, and adhered to an intensive home exercise program.

## Discussion

This case report provides an 8-year longitudinal study of a boy with spastic bilateral CP and crouch gait. The biomechanical factors contributing to our patient’s crouch gait included weak ankle plantar flexors [7], short and spastic hamstrings [3], moderately short hip flexors [4, 5], and external rotation of the tibia [8, 9]. Our patient had strong hip and knee extensors and mild to no knee flexion contractures. His selective motor control was moderately impaired on the right and mildly impaired on the left [10]. His postural balance was impaired [11], as measured by the average radial deviation and path length of the center of pressure. Although hamstring lengthening was recommended from age 7 on, he and his family opted for non-surgical treatment through a combination of BoNT-A injections and casting (Table 1), as well as physical therapy and exercise.

### Weakness

Our patient had weak plantar flexors with a 3/5 rating on manual muscle testing from ages 6 to 8, indicating the ability to do 1–9 single limb heel rises (whereas 20 heel rises would be considered normal, 5/5 strength). At age 9, his plantar flexor strength decreased to 2/5, bilaterally, indicating an inability to perform at least one single limb heel rise. Weak calf muscles have been shown to correlate with crouch gait [7], and at age 10 it appeared his weak calf muscles, in combination with an increased BMI, resulted in a worsening crouch gait. Our patient’s progressive weakness from 3/5 to 2/5 was perhaps a result of the repeated BoNT-A injections to his calf [34], which were neither indicated nor recommended by motion analysis after age 6.

### Spasticity and shortened muscle-tendon length

Our patient had 10° knee flexion contractures that contributed to his crouch gait. Further, the muscle-tendon length and lengthening velocity data for his medial hamstrings (semimembranosus) suggest his hamstrings were consistently short at initial contact and had a reduced lengthening velocity in swing (Fig. 2). A shortened semimembranosus length has been shown to correlate with increased knee flexion at initial contact and increased knee flexion in single limb stance [35], both of which contribute to a crouch gait.

### Impaired selective motor control

Our patient had moderately impaired selective motor control on the right (SCALE score = 5/10) and mildly impaired selective motor control on the left (SCALE score = 8/10). Impaired selective motor control results in abnormal movement patterns during gait, including flexion or extension synergies. During normal terminal swing, the combination of hip flexion, knee extension, and ankle dorsiflexion require selective motor control [10]. Thus, impaired selective motor control may result in coupled movement patterns involving co-activation of the quadriceps and gastrocnemius [36], as is demonstrated by our patient in Fig. 3. A lower SCALE score has been shown to correlate with an impaired ability to uncouple hip and knee movements during the swing phase of gait [10], resulting in greater knee flexion at initial contact [35]. Indeed, our patient had a lower SCALE score on his right compared to his left leg, and demonstrated greater knee flexion at initial contact on the right than the left.

### Stance-phase knee extension

Stance-phase knee extension improved from 6 to 9 years of age, worsened at age 10, improved from 11 to 12 years, and worsened again at age 13 (Fig. 1d). The increased crouch gait, as determined by maximum knee extension in stance, at age 10 was concurrent with our patient’s greatest BMI, reduced plantar flexor strength, diminished standing postural balance, slowest normalized walking speed, and greatest walking energy expenditure. His maximum knee extension in stance improved from 6 to 8 years of age, despite an increasing BMI. From 9 to 13 years of age, changes in his maximum knee extension in stance paralleled changes in his BMI (Fig. 1b). Thus, it is not clear whether BMI, in and of itself, was a critical contributor to the magnitude of crouch, as noted by Rose *et al*. [23]. Overall, maximum stance-phase knee extension improved by 14–15°, bilaterally, from age 6 to age 13.

### Swing-phase knee flexion

Swing-phase peak knee flexion was WNL from 6 to 10 years of age but declined considerably at age 11 (Fig. 2). Peak knee flexion in swing has been shown to be highly correlated to knee flexion velocity at toe-off [37], which is accomplished primarily through the action of the hip flexors and gastrocnemius [38]. Thus, our patient’s flexed hip posture and decreased plantar flexor strength likely contributed to his reduced swing-phase peak knee flexion. Our patient received BoNT-A to his rectus femoris at age 11 without a resulting change in peak knee flexion in swing.

### Progression of crouch gait

Our patient’s crouch gait worsened at age 10 despite improvements from 6 to 9 years. His maximum stance-phase knee extension at age 10 was 31°, bilaterally. As noted above, this increased crouch was concurrent with his greatest BMI and second greatest BMI-for-age percentile (Fig. 1), as well as 10° knee flexion contractures (Table 2). The increased crouch was accompanied by poor postural balance, slower than average speed when normalized for leg length, and greater than normal EEI. The energy expenditure deficits likely reflect the increased demand that stance-phase knee flexion reportedly places on the quadriceps: 210 % of body weight at 30° of knee flexion [12].

Our patient’s crouch gait varied from moderate to mild, with BoNT-A and casting once a year from ages 6 to 9 years and every 6 months from 10 to 13 years, combined with regular stretching and strengthening exercises. Non-surgical interventions for mild cases of crouch gait typically include BoNT-A injections to the hamstrings, pre-tibial bracing, and physical therapy for stretching and strengthening exercises [39], whereas soft tissue surgeries, such as hamstring or psoas lengthening, are often recommended for moderate cases of crouch gait [40–42]. Reports in the literature show that surgical lengthening of the hamstrings improve maximum knee extension in stance by an average of 11° (range 8–18°, *n* = 218 limbs) [43–45]. In our patient, a combination of regular BoNT-A with casting and physical therapy showed similar control of his crouch gait as reported for soft tissue surgery. Because maximum knee extension in single limb stance has been shown to be highly correlated to knee flexion contracture and maximum length of the semimembranosus [35], perhaps our patient’s history of BoNT-A injections with casting and physical therapy were able to halt the advancement of knee flexion contractures and/or shortening of the semimembranosus to allow for an overall, improved maximum stance-phase knee extension.

However, despite an improvement in our patient’s crouch gait at ages 11 and 12, his maximum knee extension in stance, bilateral hip flexion contractures, stride length, bilateral single limb support, and balance worsened by age 13. These functional declines may be related to the many changes, that is, physical, hormonal, and cognitive, that occur during adolescence. For example, between ages 12 and 13, both his height and weight increased at a rate greater than his average growth rate. It remains unclear how the remaining growth of adolescence will affect his gait at skeletal maturity.

### Limitations

Limitations of this case study include the lack of objective strength measures and an inability to consistently acquire gait kinetics every year due to variable participant cooperation and/or fatigue. However, when we were able to collect kinetics, two clear patterns emerged: reduced hip abductor moments during stance and reduced ankle plantar flexor moments in terminal stance, bilaterally, which were consistent with physical measures of strength (4/5 muscle strength for hip abduction and 3/5 to 2/5 muscle strength for plantar flexors).

Further, this case study is not representative of all children with spastic diplegia, and this patient’s response to treatment may not extend to other patients. Our patient was born full term, he had normal cognition, his family was able to cover all treatment costs, and he adhered to post-intervention rehabilitation and a general home exercise program. Every brain injury associated with CP is unique and, thus, individuals with CP represent a heterogeneous population. Regardless, longitudinal case studies of individuals with CP can provide a point of reference for clinicians to evaluate future patients and consider the complex interaction of treatments, growth, and external factors that influence movement in CP.

## Conclusions

As this case report highlights, the assessment and treatment of crouch gait is multifaceted. The motor deficits associated with spastic cerebral palsy, including weakness, spasticity, shortened muscle-tendon lengths, and impaired selective motor control, were identified by our patient’s clinical motion analyses, along with poor postural balance. Overall, his crouch gait improved mildly with aggressive non-operative management and a supportive family dedicated to a regular home exercise program. The annual clinical motion analyses identified changes in motor deficits that were associated with changes in the child’s walking pattern, suggesting that detailed clinical motion analyses can serve to focus treatment to improve future outcomes for children with spastic CP. When evaluating the treatment options of surgical intervention versus annual BoNT-A injections and casting, consideration must be given to the effects on muscle strength, the treatment time, financial costs, risk of repeated general anesthesia, rehabilitation requirements, and the patient’s cognitive abilities.

## References

[1] Wren TA, Rethlefsen S, Kay RM. Prevalence of specific gait abnormalities in children with cerebral palsy: influence of cerebral palsy subtype, age, and previous surgery. J Pediatr Orthop. 2005;25(1):79–83. 10.1097/00004694-200501000-00018 15614065

[2] Rodda JM, Graham HK, Carson L, et al. Sagittal gait patterns in spastic diplegia. J Bone Joint Surg (Br). 2004;86(2):251–8. 10.1302/0301-620x.86b2.13878 15046442

[3] Baumann JU, Ruetsch H, Schürmann K. Distal hamstring lengthening in cerebral palsy: an evaluation by gait analysis. Int Orthop. 1980;3(4):305–9. 10.1007/BF00266026 7399771

[4] Roosth HP. Flexion deformity of the hip and knee in spastic cerebral palsy: treatment by early release of spastic hip-flexor muscles. J Bone Joint Surg Am. 1971;53(8):1489–510. 5121791

[5] Reimers J. Static and dynamic problems in spastic cerebral palsy. J Bone Joint Surg (Br). 1973;55:822–7. 4766188

[6] Arnold AS, Anderson FC, Pandy MG, et al. Muscular contributions to hip and knee extension during the single limb stance phase of normal gait: a framework for investigating the causes of crouch gait. J Biomech. 2005;38(11):2181–9. 10.1016/j.jbiomech.2004.09.036 16154404

[7] Gage JR, Novacheck TF. An update on the treatment of gait problems in cerebral palsy. J Pediatr Orthop B. 2001;10(4):265–74. 11727367

[8] Gage JR, Schwartz MH. Pathological gait and lever-arm dysfunction. In: Gage JR, editor. The treatment of gait problems in cerebral palsy. London: Mac Keith Press; 2004. p. 180–204.

[9] Hicks JL, Arnold AS, Anderson FC, et al. The effect of excessive tibial torsion on the capacity of muscles to extend the hip and knee during single-limb stance. Gait Posture. 2007;26(4):546–52. 10.1016/j.gaitpost.2006.12.003 PMC2443695 17229573

[10] Fowler EG, Goldberg EJ. The effect of lower extremity selective voluntary motor control on interjoint coordination during gait in children with spastic diplegic cerebral palsy. Gait Posture. 2009;29:102–7. 10.1016/j.gaitpost.2008.07.007 18786827

[11] Gage JR. Treatment principles for crouch gait. In: Gage JR, editor. The treatment of gait problems in cerebral palsy. London: Mac Keith Press; 2004. p. 382–97.

[12] Perry J, Antonelli D, Ford W. Analysis of knee-joint forces during flexed-knee stance. J Bone Joint Surg Am. 1975;57(7):961–7. 1194303

[13] Rose J, Gamble JG, Medeiros J, et al. Energy cost of walking in normal children and in those with cerebral palsy: comparison of heart rate and oxygen uptake. J Pediatr Orthop. 1989;9(3):276–9. 2723046

[14] Waters RL, Mulroy S. The energy expenditure of normal and pathologic gait. Gait Posture. 1999;9(3):207–31. 10.1016/s0966-6362(99)00009-0 10575082

[15] Doralp S, Bartlett DJ. The prevalence, distribution, and effect of pain among adolescents with cerebral palsy. Pediatr Phys Ther. 2010;22(1):26–33. 10.1097/PEP.0b013e3181ccbabb 20142702

[16] Lloyd-Roberts GC, Jackson AM, Albert JS. Avulsion of the distal pole of the patella in cerebral palsy: a cause of deteriorating gait. J Bone Joint Surg (Br). 1985;67(2):252–4. 10.1302/0301-620X.67B2.3980536 3980536

[17] Opheim A, Jahnsen R, Olsson E, et al. Walking function, pain, and fatigue in adults with cerebral palsy: a 7-year follow-up study. Dev Med Child Neurol. 2009;51(5):381–8. 10.1111/j.1469-8749.2008.03250.x 19207296

[18] Steele KM, Demers MS, Schwartz MH, et al. Compressive tibiofemoral force during crouch gait. Gait Posture. 2012;35(4):556–60. 10.1016/j.gaitpost.2011.11.023 PMC3319529 22206783

[19] Campbell J, Ball J. Energetics of walking in cerebral palsy. Orthop Clin North Am. 1978;9(2):374–7. 662304

[20] Bell KJ, Ounpuu S, DeLuca PA, et al. Natural progression of gait in children with cerebral palsy. J Pediatr Orthop. 2002;22(5):677–82. 12198474

[21] Gough M, Eve LC, Robinson RO, et al. Short-term outcome of multilevel surgical intervention in spastic diplegic cerebral palsy compared with the natural history. Dev Med Child Neurol. 2004;46:91–7. 10.1017/s0012162204000192 14974633

[22] Johnson DC, Damiano DL, Abel MF. The evolution of gait in childhood and adolescent cerebral palsy. J Pediatr Orthop. 1997;17(3):392–6. 9150031

[23] Rose GE, Lightbody KA, Ferguson RG, et al. Natural history of flexed knee gait in diplegic cerebral palsy evaluated by gait analysis in children who have not had surgery. Gait Posture. 2010;31:351–4. 10.1016/j.gaitpost.2009.12.006 20116253

[24] Palisano RJ, Hanna SE, Rosenbaum PL, et al. Validation of a model of gross motor function for children with cerebral palsy. Phys Ther. 2000;80(10):974–85. 11002433

[25] Centers for Disease Control and Prevention. About BMI for children and teens. http://www.cdc.gov/healthyweight/assessing/bmi/childrens_bmi/about_childrens_bmi.html. Accessed 10 Jul 2015.

[26] Kendall FP, McCreary EK, Provance PG. Muscles: testing and function. 4th ed. Baltimore: Williams & Wilkins; 1993.

[27] Lunsford BR, Perry J. The standing heel-rise test for ankle plantar flexion: criterion for normal. Phys Ther. 1995;75(8):694–8. 10.1093/ptj/75.8.694 7644573

[28] Fowler EG, Staudt LA, Greenberg MB, et al. Selective Control Assessment of the Lower Extremity (SCALE): development, validation, and interrater reliability of a clinical tool for patients with cerebral palsy. Dev Med Child Neurol. 2009;51(8):607–14. 10.1111/j.1469-8749.2008.03186.x 19220390

[29] Schwartz MH, Rozumalski A. The Gait Deviation Index: a new comprehensive index of gait pathology. Gait Posture. 2008;28(3):351–7. 10.1016/j.gaitpost.2008.05.001 18565753

[30] Arnold AS, Liu MQ, Schwartz MH, et al. The role of estimating muscle-tendon lengths and velocities of the hamstrings in the evaluation and treatment of crouch gait. Gait Posture. 2006;23(3):273–81. 10.1016/j.gaitpost.2005.03.003 15964759

[31] Rose J, Wolff DR, Jones VK, et al. Postural balance in children with cerebral palsy. Dev Med Child Neurol. 2002;44(1):58–63. 10.1017/s0012162201001669 11811652

[32] Rose J, Gamble JG, Lee J, et al. The Energy Expenditure Index: a method to quantitate and compare walking energy expenditure for children and adolescents. J Pediatr Orthop. 1991;11(5):571–8. 1918341

[33] McDowell MA, Fryar CD, Ogden CL, et al. Anthropometric reference data for children and adults: United States, 2003–2006. Hyattsville: National Center for Health Statistics; 2008. 25585443

[34] Fortunaa R, Vazb MA, Youssefa AR, et al. Changes in contractile properties of muscles receiving repeat injections of botulinum toxin (Botox). J Biomech. 2011;44(1):39–44. 10.1016/j.jbiomech.2010.08.020 20828699

[35] Rha DW, Cahill-Rowley K, Young J, et al. Biomechanical and clinical correlates of stance-phase knee flexion in persons with spastic cerebral palsy. PM R. 2016;8(1):11–8. 10.1016/j.pmrj.2015.06.003 26079863

[36] Policy JF, Torburn L, Rinsky LA, et al. Electromyographic test to differentiate mild diplegic cerebral palsy and idiopathic toe-walking. J Pediatr Orthop. 2001;21(6):784–9. 11675555

[37] Reinbolt JA, Fox MD, Arnold AS, et al. Importance of preswing rectus femoris activity in stiff-knee gait. J Biomech. 2008;41:2362–9. 10.1016/j.jbiomech.2008.05.030 PMC5507200 18617180

[38] Fox MD, Delp SL. Contributions of muscles and passive dynamics to swing initiation over a range of walking speeds. J Biomech. 2010;43(8):1450–5. 10.1016/j.jbiomech.2010.02.009 PMC2866832 20236644

[39] Gormley Jr ME. Treatment of neuromuscular and musculoskeletal problems in cerebral palsy. Pediatr Rehabil. 2001;4(1):5–16. 10.1080/13638490151068393 11330850

[40] Novacheck TF, Trost JP, Schwartz MH. Intramuscular psoas lengthening improves dynamic hip function in children with cerebral palsy. J Pediatr Orthop. 2002;22:158–64. 11856921

[41] Bleck EE. Orthopaedic management in cerebral palsy. London: Mac Keith Press; 1987.

[42] Root L. Distal hamstring surgery in cerebral palsy. In: Sussman MD, editor. The diplegic child: evaluation and management. Rosemont: American Academy of Orthopaedic Surgeons; 1992. p. 309–36.

[43] Chang W-N, Tsirikos AI, Miller F, et al. Distal hamstring lengthening in ambulatory children with cerebral palsy: primary versus revision procedures. Gait Posture. 2004;19:298–304. 10.1016/S0966-6362(03)00070-5 15125919

[44] DeLuca PA, Õunpuu S, Davis III RB, et al. Effect of hamstring and psoas lengthening on pelvic tilt in patients with spastic diplegic cerebral palsy. J Pediatr Orthop. 1998;18(6):712–8. 9821124

[45] Õunpuu S, Muik E, Davis III RB, et al. Rectus femoris surgery in children with cerebral palsy. Part I: the effect of rectus femoris transfer location on knee motion. J Pediatr Orthop. 1993;13:325–30. 10.1097/01241398-199305000-00010 8496366

